# Nociceptive Response Is a Possible Marker of Evolution in the Level of Consciousness in Unresponsive Wakefulness Syndrome Patients

**DOI:** 10.3389/fnins.2021.771505

**Published:** 2021-12-15

**Authors:** Maria Daniela Cortese, Francesco Arcuri, Idan E. Nemirovsky, Lucia Francesca Lucca, Paolo Tonin, Andrea Soddu, Francesco Riganello

**Affiliations:** ^1^Research in Advanced Neurorehabilitation (RAN), S. Anna Institute, Via Siris, Crotone, Italy; ^2^Department of Physics and Astronomy, Brain and Mind Institute, Western University, London, ON, Canada

**Keywords:** disorders of consciousness, nociception coma scale, nociception coma scale revised, coma recovery scale-revised, pain, vegetative state, unresponsive wakefulness syndrome, minimally conscious state

## Abstract

The Nociception Coma Scale (NCS) and its revised version (NCS-R) were used to evaluate behavioral responses to pain in non-communicative patients. We hypothesized that if patients demonstrate changes to their NCS(-R) scores over time, their evolving behavioral abilities could indicate a forthcoming diagnostic improvement with the Coma Recovery Scale-Revised (CRS-R). Forty-three Vegetative State/Unresponsive Wakefulness Syndrome (VS/UWS) patients were enrolled in the study. The patients were assessed weekly using the CRS-R and NCS(-R) for four consecutive weeks. The first assessment was within 10 days after hospitalization. The assessments were performed between 09:30 and 11:30 AM in a room with constant levels of humidity, light and temperature, as well as an absence of transient noise. Noxious stimuli were administered using a Newton-meter, with pressure applied to the fingernail bed for a maximum of 5 s unless interrupted by a behavioral response from subjects. Seventeen patients demonstrated improvements in their level of consciousness, 13 of whom showed significant behavioral changes through the NCS(-R) before being diagnosed with a Minimally Conscious State (MCS) according to the CRS-R. The behavioral changes observed using the NCS(-R) corresponded to a high probability of observing an improvement from VS/UWS to MCS. To characterize the increased likelihood of this transition, our results present threshold scores of ≥5 for the NCS (accuracy 86%, sensitivity 87%, and specificity 86%) and ≥3 for the NCS-R (accuracy 77%, sensitivity 89%, and specificity 73%). In conclusion, a careful evaluation of responses to nociceptive stimuli in DOC patients could constitute an effective procedure in assessing their evolving conscious state.

## Introduction

One of the most important challenges in caring for patients with Disorders of Consciousness (DOC) is to correctly classify their severity by differentiating the two most misdiagnosed conditions: the Vegetative State/Unresponsive Wakefulness Syndrome (VS/UWS) and the Minimally Conscious State (MCS).

In the VS/UWS condition, there is no behavioral evidence of self or environmental awareness. However, behavioral sleep/wake cycles and arousal are preserved (Multi-Society Task Force on PVS, 1994). In contrast, MCS patients show some signs of awareness, such as visual pursuit, localization to pain, or non-systematic command-following, though they are unable to communicate their thoughts or feelings (Giacino and Kalmar, 2005; Giacino and Smart, 2007).

It is estimated that the misdiagnosis rate for DOC populations falls within the range of 30 to 45% (Schnakers et al., 2009) when they are assessed without standardized neurobehavioral tools (Seel et al., 2010; Wang et al., 2020).

The current “Gold-Standard” for behavioral scales, known as the Coma Recovery Scale-Revised (CRS-R), was developed with the purpose of differentiating the MCS and VS/UWS conditions (Giacino et al., 2004). However, even with its higher psychometric standards, CRS-R scoring may be influenced by a subjective interpretation of observed behaviors (Cortese et al., 2014).

As noted, the ability to orient attention toward a painful stimulus is generically preserved in MCS patients (de Tommaso et al., 2015). Nociception describes the decoding of a potentially tissue-damaging event by nociceptors (Loeser and Treede, 2008), which tends to be followed by a response of the Autonomic Nervous System (ANS) (Kyle and McNeil, 2014; Mischkowski et al., 2018). Nociceptive stimulation is transmitted through the spinothalamic tract to reach the thalamus and the cortex (Loeser and Treede, 2008; Morton et al., 2016), eliciting the activation of an expansive cortical network (Coghill et al., 2003; Chatelle et al., 2014). While reflex responses are said to be modulated by the midbrain and thalamus (Morton et al., 2016), the sensory–discriminative dimension of pain processing involves higher order cortical areas such as the secondary somatosensory (S2) cortex and the posterior insula (lateral network) (Ploner et al., 2002; Lockwood et al., 2013).

Neuroimaging studies have so far provided important knowledge on the different pain processing capabilities of DOC patients. However, the ability of the VS/UWS population to properly process pain remains unclear (Chatelle and Thibaut, 2014). Compared to healthy controls, the S1 cortex of VS/UWS patients was found to be functionally disconnected from the S2, bilateral posterior parietal, premotor, polysensory superior temporal, and prefrontal cortices (Boly et al., 2008) when noxious stimuli were administered. In contrast, MCS patients showed brain activation patterns that were similar to controls (Boly et al., 2008), with the Insula and Anterior Cingulate Cortex (ACC) activated, suggesting a greater likelihood that MCS patients perceive pain (Shackman et al., 2011).

In evaluating different states of consciousness, an examiner can gain valuable insight by observing behavioral responses to nociceptive stimulation. To achieve this, Schnakers et al. (2010) developed the Nociception Coma Scale (NCS) that specifically evaluates a range of behaviors linked to pain in VS/UWS and MCS patients. The NCS consists of 4 subscales assessing motor, verbal, facial and visual responses. Chatelle and colleagues proposed a revised version (NCS-R) (Chatelle and Thibaut, 2014), which excluded the visual subscale.

This study aims to verify if an improvement in the NCS(-R) score over multiple assessments could correspond to an improvement in a patient’s condition as indicated by the CRS-R.

We hypothesized that by observing gradual changes in the NCS(-R) total scores of patients, there may be an identifiable threshold score that indicates a higher probability of improved outcomes for patients, and more specifically to the patients in our study, a transition from a VS/UWS to a MCS diagnosis.

## Materials and Methods

In this study, we enrolled patients who have been hospitalized at the S. Anna institute of Crotone (Italy) for 3 years and diagnosed as VS/UWS based on the Aspen Workgroup criteria (Giacino et al., 2004; Table 1). Patients were excluded from the study for the following: (i) documented history of prior brain injury; (ii) premorbid history of developmental, psychiatric or neurologic illness resulting in documented functional disability up to the time of the injury; (iii) neurological or psychiatric disease history; (iv) upper limb contusions, fractures, or flaccid paralysis; (v) mechanical ventilation; (vi) clinical instability, including treatment with neuroactive drugs, and concurrent systemic disorders, or evidence of recurrent pain as assessed by clinicians (e.g., alert response to tactile stimuli, facial expressions of pain); (vii) a transition out of VS/UWS diagnosis within 2 weeks of the first CRS-R and NCS(-R) assessments.

**TABLE 1:** Demographics information.

VS/UWS°(VS/UWS→VS/UWS)					VS/UWS^1^ (VS/UWS→MCS)				
subj	Gender	Age	Aetiology	time from injury (days)	subj	Gender	Age	Aetiology	time from injury (days)
8	male	64–74	TBI	41	13	male	62–81	HEM	52
17	male		HEM	37	22	male		TBI	45
30	male		HEM	44	42	male		TBI	25
31	male		Anox	40	43	male		HEM	20
1	male	56–63	HEM	54	18	male	47–56	Other	25
5	male		Anox	52	19	male		HEM	40
3	male		TBI	25	20	male		HEM	27
4	male		TBI	28	35	male		TBI	32
12	male		TBI	39	37	male		TBI	41
26	male		HEM	35	9	male	27–51	TBI	39
10	male	19–54	TBI	40	15	male		HEM	37
16	male		Anox	45	38	male		TBI	23
21	male		TBI	35	7	female	32–65	TBI	35
25	male		Anox	41	14	female		HEM	34
27	male		TBI	29	33	female		TBI	37
39	male		Anox	40	34	female		TBI	25
41	male		HEM	75	36	female		TBI	32
2	female	58–75	HEM	28					
23	female		HEM	55					
24	female		HEM	45					
40	female		HEM	72					
6	female	39–55	HEM	40					
11	female		Anox	32					
28	female		HEM	45					
29	female		Anox	37					
32	female		HEM	51					

### Participants

Patients were enrolled within 10 days after hospitalization. With the exclusion criteria, 43 patients were included in the study (29 males, age 54 ± 14, 10 hemorrhagic, 13 traumatic, 5 cardiac arrests and 1 other etiology, time from injury 38 ± 11 days; 14 females, age 53 ± 12, 8 hemorrhagic, 4 traumatic and 2 cardiac arrests, time from injury 41 ± 12 days (Table 1).

The study was carried out following the rule of the Declaration of Helsinki^1^, approved by the Ethic Committee of “Regione Calabria Comitato Etico Sezione Area Centro” of Catanzaro. Written informed consent was obtained by the patients’ legal representatives.

### Procedure

All patients were enrolled within the first 10 days of hospitalization if diagnosed as VS/UWS during clinical assessments. Then, they were assessed with the CRS-R and NCS(-R) every week for four consecutive weeks (Tables 2, 3). In the CRS-R, scoring is based on the presence or absence of specific behavioral responses to sensory stimuli that are administered in a standardized manner, resulting in a total score between 0 and 23. On the other hand, the NCS consists of four subscales that assess motor, verbal, visual, and facial responses. Each subscale ranges from 0 (no response) to 3 (appropriate response), for a total score that ranges from 0 to 12. The NCS-R does not include the former assessment’s visual subscale and thus has a score ranging from 0 to 9.

**TABLE 2:** Behavioral assessment of the patients that non-change the level of consciousness.

					CRS-R						NCS				NCS-R
**Subject**	**Time_from_injury**	**Sequence of rec.**	**Auditory**	**Visual**	**Motor**	**Oromotor**	**Communication**	**Arousal**	**Total**	**Motor**	**Verbal**	**Facial**	**Visual**	**Total**	**Total**

1	54	0	0	0	1	1	0	0	2	2	0	0	1	3	2
		1	0	0	2	1	0	0	3	2	0	0	1	3	2
		2	1	0	1	1	0	2	5	1	0	1	1	3	2
		3	2	1	2	1	0	2	8	1	0	1	1	3	2
2	28	0	1	0	2	1	0	1	5	1	0	1	0	2	2
		1	1	0	2	1	0	1	5	2	0	1	0	3	3
		2	1	0	2	1	0	2	6	2	0	1	0	3	3
		3	1	0	2	1	0	1	5	2	0	2	0	4	4
3	25	0	1	0	1	1	0	1	4	2	0	0	0	2	2
		1	1	0	2	1	0	1	5	2	0	0	1	3	2
		2	1	0	2	1	0	1	5	2	0	0	1	3	2
		3	1	0	2	1	0	1	5	2	0	0	1	3	2
4	28	0	1	0	2	1	0	1	5	2	0	0	1	3	2
		1	1	0	2	1	0	0	4	2	0	0	1	3	2
		2	2	1	2	1	0	2	8	2	0	0	1	3	2
		3	2	1	2	1	0	1	7	2	0	1	2	5	3
5	52	0	1	0	1	1	0	1	4	2	0	0	0	1	2
		1	1	0	1	1	0	2	5	1	0	0	0	1	1
		2	1	0	1	1	0	2	5	1	0	1	0	2	2
		3	1	0	1	1	0	1	4	1	0	1	0	2	2
6	40	0	1	0	1	1	0	0	3	1	0	0	0	1	1
		1	1	0	1	1	0	0	3	1	0	0	0	1	1
		2	1	0	2	1	0	1	5	1	0	0	2	3	1
		3	1	0	2	1	0	1	5	1	0	0	2	3	1
8	41	0	1	0	2	1	0	1	5	2	0	0	0	2	2
		1	1	0	2	1	0	1	5	2	0	0	0	2	2
		2	1	0	1	1	0	1	4	1	0	1	1	3	2
		3	1	0	2	1	0	1	5	2	0	0	0	2	2
10	40	0	1	0	1	0	0	2	4	1	0	0	0	1	1
		1	1	0	1	0	0	2	4	1	0	0	0	1	1
		2	1	0	1	0	0	2	4	1	0	0	0	1	1
		3	1	1	2	1	0	2	7	2	0	1	0	3	3
11	32	0	1	0	2	1	0	1	5	2	0	0	0	2	2
		1	1	0	2	1	0	2	6	2	0	0	1	3	2
		2	1	0	2	0	0	1	4	2	0	0	0	2	2
		3	1	0	2	1	0	2	6	2	0	0	1	3	2
12	39	0	2	1	1	1	0	2	7	1	1	1	1	4	3
		1	2	1	1	1	0	2	7	1	1	1	1	4	3
		2	2	1	1	1	0	2	7	1	1	1	2	5	3
		3	2	1	1	1	0	2	7	1	0	1	2	4	2
16	45	0	1	0	2	1	0	2	5	2	0	0	1	3	2
		1	1	0	2	1	0	1	5	2	0	0	1	3	2
		2	1	0	2	1	0	1	5	2	0	0	2	4	2
		3	1	1	2	1	0	2	7	2	0	1	2	5	3
17	37	0	1	0	2	1	0	1	5	1	0	1	1	3	2
		1	1	0	2	1	0	2	6	2	0	1	1	4	3
		2	1	0	2	1	0	2	6	2	0	1	1	4	3
		3	1	0	1	1	0	1	4	1	0	0	1	2	1
21	35	0	1	0	2	1	0	2	6	2	0	1	1	4	3
		1	1	0	2	1	0	2	6	2	0	1	1	4	3
		2	1	0	2	1	0	2	6	2	0	1	1	4	3
		3	1	0	2	1	0	1	5	2	0	1	1	4	3
23	55	0	0	1	2	1	0	2	6	2	0	1	1	4	3
		1	0	1	2	1	0	2	6	2	0	1	1	4	3
		2	0	1	2	1	0	2	6	2	0	2	1	5	4
		3	1	0	2	1	0	2	6	2	0	2	1	5	4
24	45	0	1	0	2	1	0	2	6	2	0	1	1	4	3
		1	1	0	2	1	0	2	6	2	0	1	1	4	3
		2	1	0	2	1	0	2	6	2	0	1	1	4	3
		3	1	0	2	1	0	2	6	2	0	1	1	4	3
25	41	0	1	1	1	0	0	2	5	1	0	0	1	2	1
		1	1	1	1	0	0	2	5	1	0	0	1	2	1
		2	1	1	1	0	0	2	5	1	0	0	1	2	1
		3	1	1	1	0	0	2	5	1	0	0	1	2	1
26	35	0	1	0	2	1	0	1	5	1	0	0	1	2	1
		1	1	0	2	1	0	2	6	1	0	0	1	2	1
		2	1	0	2	1	0	1	5	1	0	0	1	2	1
		3	1	0	2	1	0	2	6	1	0	0	1	2	1
27	29	0	2	1	2	1	0	1	7	2	2	1	0	5	5
		1	2	1	2	1	0	2	8	2	2	1	0	5	5
		2	2	1	2	1	0	1	7	2	2	1	0	5	5
		3	2	1	2	1	0	1	7	2	1	1	0	4	4
28	45	0	1	0	1	1	0	1	4	1	0	1	0	2	2
		1	1	0	1	1	0	1	4	1	0	1	0	2	2
		2	1	0	1	1	0	1	4	1	0	1	0	2	2
		3	1	0	1	1	0	1	4	1	0	1	0	2	2
29	37	0	1	1	2	1	0	1	4	2	0	1	2	5	3
		1	1	1	2	1	0	1	6	2	0	1	2	5	3
		2	1	1	2	1	0	1	6	2	0	1	2	5	3
		3	1	1	2	1	0	2	7	2	0	1	2	5	3
30	44	0	2	1	1	1	0	2	7	1	0	1	2	4	2
		1	2	1	1	1	0	2	7	1	0	1	2	4	2
		2	2	1	1	1	0	2	7	1	0	1	2	4	2
		3	2	1	1	1	0	2	7	1	0	1	2	4	2
31	40	0	1	0	1	1	0	1	4	1	0	1	1	3	2
		1	1	0	1	1	0	1	4	1	0	0	1	2	1
		2	1	1	1	1	0	1	5	1	0	1	1	3	2
		3	1	1	1	1	0	2	6	1	0	1	1	3	2
32	51	0	1	0	2	1	0	0	4	2	0	0	1	3	2
		1	1	0	2	1	0	0	4	2	0	0	1	3	2
		2	0	0	2	1	0	0	3	2	0	0	1	3	2
		3	0	0	2	1	0	0	3	2	0	0	0	2	2
39	40	0	1	0	1	1	0	2	5	1	0	1	0	2	2
		1	2	0	1	1	0	2	6	1	0	1	0	2	2
		2	2	0	1	1	0	2	6	1	0	1	0	2	2
		3	2	0	1	1	0	2	6	1	0	1	0	2	2
40	72	0	0	0	2	1	0	1	4	2	0	0	1	3	2
		1	0	0	2	1	0	1	4	2	0	1	1	4	3
		2	0	0	2	1	0	1	4	2	0	1	1	4	3
		3	0	0	2	1	0	2	5	2	0	1	1	4	3
41	75	0	0	0	2	1	0	2	5	2	0	0	1	3	2
		1	0	0	2	1	0	2	5	2	0	0	1	3	2
		2	0	0	2	1	0	2	5	2	0	1	1	4	3
		3	1	0	2	1	0	2	6	2	0	1	1	4	3

**TABLE 3:** Behavioral assessment of the patients that change the level of consciousness.

			CRS-R							NCS					NCS-R
Subject	Time_from_injury	Sequence of rec.	Auditory	Visual	Motor	Oromotor	Communication	Arousal	Total	Motor	Verbal	Facial	Visual	Total	Total
7	35	0	1	0	2	1	0	1	5	1	0	0	1	2	1
		1	1	1	2	0	0	1	5	2	0	0	0	2	2
		2	1	1	2	1	0	2	7	3	0	2	2	7	5
		**3**	**3**	**1**	**2**	**2**	**0**	**1**	**9**	**2**	**2**	**0**	**1**	**5**	**4**
9	39	0	1	0	2	1	0	1	5	2	0	1	0	3	3
		1	2	0	2	1	0	1	6	2	0	1	0	3	3
		2	1	0	2	1	0	1	5	2	0	2	1	5	4
		**3**	**4**	**5**	**1**	**1**	**0**	**2**	**13**	**1**	**0**	**3**	**0**	**4**	**4**
13	52	0	0	0	2	1	0	1	4	2	0	1	1	4	3
		1	0	0	2	1	0	1	4	2	0	1	1	4	3
		2	2	0	2	1	0	2	7	2	0	0	1	3	2
		**3**	**2**	**3**	**2**	**1**	**0**	**2**	**10**	**2**	**0**	**0**	**1**	**3**	**2**
14	34	0	1	1	2	1	0	1	6	2	1	1	1	5	4
		1	1	1	2	1	0	2	7	2	1	1	2	6	4
		**2**	**2**	**1**	**2**	**2**	**0**	**2**	**9**	**2**	**1**	**1**	**2**	**6**	**4**
		**3**	**2**	**1**	**2**	**2**	**0**	**2**	**9**	**2**	**0**	**1**	**2**	**5**	**3**
15	37	0	1	1	2	1	0	1	6	2	0	0	0	2	2
		1	1	1	2	1	0	1	6	2	0	0	0	2	2
		**2**	**2**	**2**	**2**	**1**	**0**	**2**	**9**	**2**	**0**	**1**	**0**	**3**	**3**
		**3**	**2**	**3**	**1**	**1**	**0**	**2**	**9**	**1**	**0**	**2**	**2**	**5**	**3**
18	25	0	1	2	2	1	0	1	7	2	1	1	2	6	4
		1	1	2	2	1	0	2	8	2	2	1	2	7	5
		2	1	1	2	2	0	2	8	2	2	1	2	7	5
		**3**	**3**	**3**	**1**	**2**	**0**	**2**	**11**	**1**	**2**	**1**	**2**	**6**	**4**
19	40	0	0	3	1	1	0	1	6	1	0	1	1	3	2
		1	0	3	1	1	0	1	6	1	0	1	0	2	2
		**2**	**3**	**3**	**1**	**2**	**1**	**2**	**12**	**1**	**0**	**1**	**1**	**3**	**2**
		**3**	**2**	**3**	**1**	**1**	**0**	**2**	**9**	**1**	**0**	**1**	**1**	**3**	**2**
20	27	0	2	2	1	1	0	2	8	1	0	1	1	3	2
		1	2	2	1	1	0	2	8	1	0	1	1	3	2
		2	1	1	2	2	0	2	8	2	2	1	2	7	5
		**3**	**2**	**0**	**5**	**2**	**0**	**2**	**11**	**2**	**2**	**1**	**2**	**7**	**5**
22	45	0	1	0	2	1	0	1	5	2	0	0	1	3	2
		1	1	0	2	1	0	1	5	2	0	0	1	3	2
		**2**	**4**	**0**	**3**	**1**	**0**	**2**	**10**	**3**	**0**	**2**	**1**	**6**	**5**
		**3**	**3**	**0**	**3**	**1**	**0**	**2**	**9**	**3**	**0**	**2**	**1**	**6**	**5**
33	37	0	2	1	2	1	0	1	7	2	0	0	2	4	2
		1	2	1	2	1	0	1	7	3	0	0	2	5	3
		**2**	**4**	**1**	**2**	**2**	**0**	**1**	**10**	**2**	**2**	**1**	**2**	**7**	**5**
		**3**	**4**	**3**	**2**	**2**	**2**	**1**	**11**	**2**	**0**	**2**	**2**	**6**	**4**
34	25	0	2	1	2	1	0	1	7	2	0	1	2	5	3
		1	2	1	2	1	0	1	7	2	0	1	2	5	3
		**2**	**4**	**3**	**2**	**1**	**0**	**1**	**11**	**2**	**0**	**3**	**1**	**6**	**5**
		**3**	**4**	**4**	**2**	**1**	**0**	**1**	**13**	**2**	**0**	**3**	**1**	**6**	**5**
35	32	0	2	0	1	1	0	1	5	1	0	1	0	2	2
		1	2	0	1	1	0	1	5	1	0	1	0	2	2
		2	2	0	2	1	0	2	7	2	0	3	0	5	5
		**3**	**3**	**0**	**3**	**1**	**0**	**2**	**9**	**3**	**0**	**3**	**0**	**6**	**6**
36	32	0	1	1	1	2	0	1	6	1	0	2	2	5	3
		1	1	1	1	2	0	1	6	1	0	2	2	5	3
		**2**	**1**	**1**	**5**	**2**	**0**	**1**	**10**	**2**	**0**	**2**	**2**	**6**	**4**
		**3**	**1**	**1**	**5**	**2**	**0**	**1**	**10**	**2**	**0**	**3**	**2**	**7**	**5**
37	41	0	0	0	2	1	0	2	5	2	0	0	1	3	2
		1	2	1	2	1	0	2	8	2	1	2	2	7	5
		2	1	0	2	2	0	2	7	2	1	2	2	7	5
		**3**	**2**	**2**	**2**	**1**	**1**	**2**	**10**	**2**	**0**	**1**	**2**	**6**	**4**
38	23	0	1	1	2	0	0	1	5	2	0	1	2	5	4
		1	1	1	2	0	0	1	5	2	0	1	2	5	4
		2	2	1	2	0	0	1	6	2	0	1	2	5	4
		**3**	**3**	**3**	**1**	**1**	**0**	**2**	**10**	**2**	**1**	**2**	**2**	**7**	**5**
42	25	0	1	1	2	0	0	1	5	2	0	1	2	5	3
		1	1	1	2	0	0	1	5	2	0	1	2	5	3
		**2**	**3**	**3**	**0**	**1**	**0**	**2**	**9**	**2**	**1**	**2**	**1**	**6**	**5**
		**3**	**3**	**3**	**1**	**1**	**0**	**2**	**10**	**2**	**2**	**3**	**2**	**9**	**7**
43	20	0	1	0	2	2	0	1	6	2	0	1	2	5	3
		1	1	0	2	2	0	1	6	2	0	1	2	5	3
		**2**	**3**	**3**	**3**	**1**	**0**	**2**	**12**	**3**	**0**	**1**	**2**	**6**	**4**
		**3**	**3**	**2**	**4**	**1**	**0**	**2**	**13**	**3**	**0**	**2**	**2**	**7**	**5**

*In bold the assessed MCS condition.*

All patients were nursed for at least 30 min prior to the protocol to avoid any external interference. The assessments were performed between 9:30 AM and 11:30 AM to maximize the probability of observing responses to stimuli (Cortese et al., 2014; Giacino et al., 2018).

Noxious stimuli were administered with a Newton-meter (Force Dial, FDN 200 model; Connecticut, United States^2^) following the procedure described by NCS-R guidelines. This consisted of applying pressure on the fingernail bed for a maximum of 5 s, unless interrupted by a behavioral response from the patient.

The stimuli were administered in a lab with conditions of constant temperature (24°C), humidity, and luminosity. It was important to ensure the absence of transient noise, as well as to avoid any influence from nursing, feeding, or rehabilitative programs.

The scales were administered by two expert examiners. To avoid the impact of one evaluation on the next, the examiner was chosen randomly for the first assessment. Subsequent assessments were performed with alternation between the two examiners, who were unaware of previous CRS-R and NCS(-R) scores.

### Statistical Analysis

For the analysis, we divided patients into two groups: VS/UWS patients who underwent a change in their level of consciousness (VS/UWS^1^) and improved to MCS, and those who did not (VS/UWS°).

The time at which a patient was diagnosed as MCS with CRS-R was denoted as *time*_0_. The scales administered at this time were referred to as CRS-R*_*time*0_*, NCS*_*time*0_*, and NCS-R*_*time*0_* (Table 2).

The times elapsed between the injury and the first behavioral assessments were compared for the two groups using the Mann-Whitney’s exact test.

**FIGURE 1:**
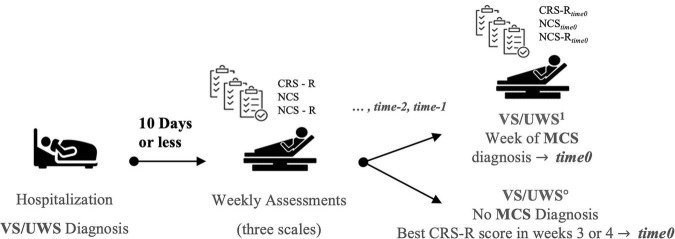
Protocol for the assessing patients. The week in which the patient is assessed as MCS is marked “time 0.” The scales’ results are compared with the two previous weeks’ results, marked as “time-1” and “time-2.”

The total score of each scale at *time*_0_ was compared to that of the two previous assessments (*time_–1_ –* 1 week prior, and *time_–2_ –* 2 weeks prior) with the Wilcoxon Signed-rank exact test. For patients who did not emerge out of VS/UWS, the highest of the CRS-R scores recorded in the third and fourth weeks was used to designate *time*_0_ (Figure 1).

The VS/UWS^1^ and VS/UWS° groups were compared in terms of the CRS-R, NCS, and NCS-R scores at *time_0_, time_–1_* and *time_–2_* using Mann-Whitney’s exact test, which is considered to be a valid choice for a small patient sample (Siegel, 1956; Gibbons and Chakraborti, 2011) or when working with sparse or unbalanced data (Tanizaki, 1997; Mundry and Fischer, 1998; Gibbons and Chakraborti, 2011).

The effect size r was calculated as the absolute value of Z/√(N), where Z was the Z-statistic and N was the total number of subjects. The different effect sizes obtained were classified as follows: insignificant if r < 0.1; low if 0.1 ≤ r < 0.3; medium if 0.3 ≤ r < 0.5; and high if r ≥ 0.5 (Rosenthal, 1991). The correlation between CRS-R and NCS was tested using the Spearman correlation test, with significance set to *p* ≤ 0.05.

Based on CRS-R assessments in which patients were first diagnosed as MCS (CRS-R*_*time*0_*), as well as NCS_*time–*1_ and NCS-R_*time–*1_ scores, a receiver operating characteristic (ROC) curve was used to define threshold NCS(-R) scores that can predict a forthcoming MCS diagnosis (i.e., whether patients will transition to MCS in the following week). These threshold scores were found using the area under the ROC curve, which was considered to have acceptable accuracy if between 0.7 and 0.8, and beyond satisfactory accuracy if between 0.8 and 0.9 (Mandrekar, 2010).

After ROC curves were used to define threshold scores that work best for prediction, we employed logistic regression to determine how well NCS and NCS-R correspond to a patient’s evolving conscious state (as defined by CRS-R). Logistic regression was first performed for the entire dataset, where we obtained the overall accuracy, sensitivity, specificity, positive and negative prediction values, and likelihood ratios for the nociceptive scales. To further validate this analysis, logistic regression was then repeated with the dataset split into training and test sets.

## Results

**FIGURE 2:**
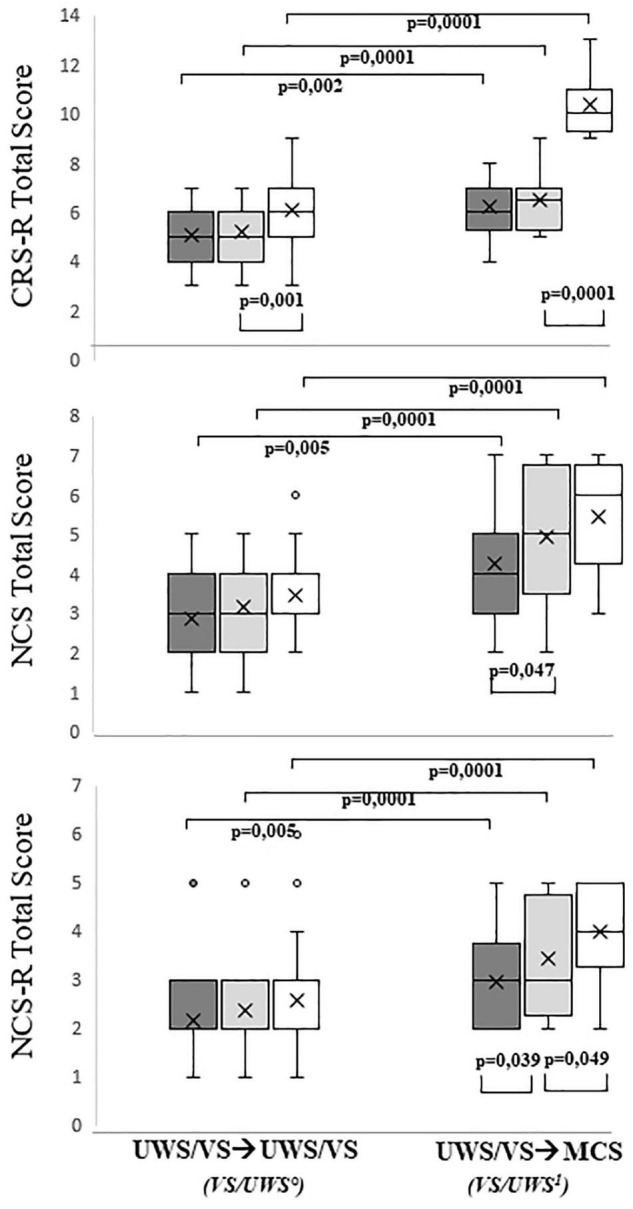
Boxplot of CRS-R, NCS, and NCS-R in time-2 (dark gray), time-1 (light gray), and time0 (white). The left column shows patients that did not change in their level of consciousness (VS/UWS°), and right column patients that transitioned to MCS (VS/UWS^1^).

Significant differences were found between the VS/UWS° and VS/UWS^1^ groups with Mann-Whitney’s exact test when comparing the time elapsed from injury (Z = −2.640, *p* = 0.007, r = 0.41). We also found significant differences between their scores at *time*_0_, *time_–1_*, and *time_–2_* for all the scales. The Mann-Whitney statistics for the tests at these times ranged as follows: CRS-R: −5.552 ≤ Z ≤ −2.756; 0.002 ≤ *p* ≤ 0.0001; 0.42 ≤ r ≤ 0.84, NCS: 4.416 ≤ Z ≤ −2.529; 0.0001 ≤ *p* ≤ 0.005; 0.34 ≤ r ≤ 0.67, and NCS-R: −4.095 ≤ Z ≤ −2.571; 0.0001 ≤ *p* ≤ 0.005; 0.35 ≤ r ≤ 0.62 (Figure 2).

Further significant differences were found using Wilcoxon’s exact within the VS/UWS^1^ group when comparing the results of a particular test at different times: NCS*_*time–*2_* and NCS*_time–1_*: Z = −1.843; *p* = 0.047; r = 0.33, NCS-R*_*time–*2_* and NCS-R*_time–1_*: Z = −1.994; *p* = 0.039; r = 0.35, NCS-R*_*time–*1_* and NCS-R*_time0_*: Z = −1.706; *p* = 0.049; r = 0.30, and CRS-R*_time–1_* and CRS-R*_time0:_* Z = −3.541; *p* = 0.0001; r = 0.63.

For VS/UWS° patients, significant differences were found using Wilcoxon’s exact test when comparing CRS-R*_*time–*1_* and CRS-R*_*time*0_* (Wilcoxon’s test Z = −2.914, *p* = 0.001, r = 0.40) (Figure 2).

In terms of their predictive capacity (i.e., accuracy of detecting a CRS-R diagnosis of MCS 1 week prior), both NCS*_*time–*1_* and NCS-R*_*time–*1_* assessments showed promising results: the ROC curve areas were 0.84 (95% CI: [0.70–0.97]) for NCS*_*time–*1_* and 0.81 (95% CI: [0.67–0.94]) for NCS-R*_*time–*1_*. The highest accuracy was obtained with a threshold of 5 for NCS and 3 for NCS-R. Using these thresholds, we obtained accuracies of 86% (76% sensitivity, 92% specificity) for the NCS and 70% (58% sensitivity and 84% specificity) for the NCS-R (Table 3).

For the NCS and NCS-R assessments at *time_–1_*, logistic regression was applied to predict the probability of observing a change in diagnosis. The regressors used were the NCS*_*time–*1_* and NCS-R*_*time–*1_* total scores, sex, age, time from injury, and etiology. From these, only NCS*_*time–*1_* and NCS-R*_*time–*1_* were found to be significant for the correct classification of patients (NCS*_*time–*1_*: *p* = 0.02; C.I. for Exp(B) [1.519–6.129]; sex, age, time from injury, and etiology 0.17 ≤ *p* ≤ 0.74; NCS-R*_*time–*1_*: *p* = 0.03; C.I. for Exp(B) [1.553–8.125]; sex, age, time from injury, and etiology 0.12 ≤ *p* ≤ 0.84).

**FIGURE 3:**
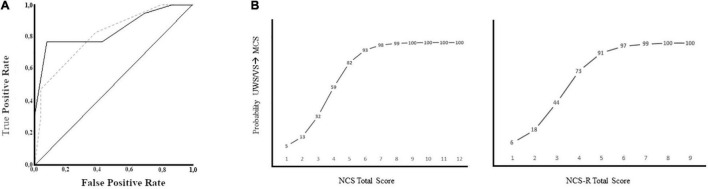
Classifications based on ROC and Logistic Regression. **(A)** The Receiver Operating Characteristic curve for NCS time-1 (continuous line) and NCS-Rtime-1 (dashed line). **(B)** Logistic classification, showing % probability (numbers on the curve) of observing a change in the consciousness level vs. the total score of the NCS (left) and NCS-R (right).

In the logistic regression, the NCS*_*time–*1_* regressor classified 86% of patients correctly (Homer-Lemeshow *p* = 0.06; Cox & Snell R^2^ = 0.34) with a sensitivity of 87%, a specificity of 86%, and positive and negative likelihood ratios of 6.1 and 0.2, respectively. For NCS-R*_*time–*1_*, 77% of the patients were correctly classified (Homer-Lemeshow *p* = 0.56; Cox & Snell R^2^ = 0.29) with a sensitivity of 89%, a specificity of 73%, and positive and negative likelihood ratios of 3.4 and 0.2, respectively (Figure 3 and Table 4).

**TABLE 4:** Confusion matrix and classification’s results by logistic regression and ROC curve.

		Logistic regression (−4.902 + 1.513×NCS)		Logistic regression (−4.056 + 1.263×NCS-R)		ROC max accuracy ≥ 5		ROC max accuracy ≥ 3	
		NCS		NCS-R		NCS		NCS-R	
		VS/UWS → MCS	VS/UWS → VS/UWS°	VS/UWS → MCS	VS/UWS → VS/UWS°	VS/UWS → MCS	VS/UWS → VS/UWS°	VS/UWS → MCS	VS/UWS → VS/UWS°
**Metrics**	**VS/UWS → MCS**	**13**	**4**	**8**	**9**	**13**	**4**	**14**	**3**
	**VS/UWS → VS/UWS°**	**2**	**24**	**1**	**25**	**4**	**22**	**10**	**16**

Bad prediction (%)		14		23		19		30	
Correct prediction (%)		86		77		81		70	
Precision (%)		76		47		76		82	
Negative predictive values (%)		92		96		85		61	
Sensitivity (%)		87		89		76		58	
Specificity (%)		86		73		85		84	
Accuracy (%)		86		77		81		70	
Balanced accuracy (%)		84		72		80		72	
positive Likelihood Ratio		6.1		3.4		5		3.7	
negative likelihood Ratio		0.2		0.2		0.3		0.5	

*There were no significant differences between the NCS and NCS-R in the confusion matrix of the classifications (Table 3), which was obtained using the ROC curve’s logistic regression and maximum accuracy (Fisher’s exact test: p ≥ 0.29).*

To validate these predictive accuracies, the dataset was split into training (*N* = 28) and test (*N* = 15) sets. As before, the only significant regressors in training were NCS*_*time–*1_* and NCS-R*_*time–*1_* (NCS*_*time–*1_*: *p* = 0.009; C.I. for Exp(B) [1.443–12.514]; sex, age, time from injury, and etiology 0.15 ≤ *p* ≤ 0.68; NCS-R*_*time–*1_*: *p* = 0.01; C.I. for Exp(B) [1.521–21.646]; sex, age, time from injury, and etiology 0.24 ≤ *p* ≤ 0.78).

With the NCS*_*time–*1_* training scores (*N* = 28), the procedure classified 89% of patients correctly (Homer-Lemeshow *p* = 0.253; Cox & Snell R^2^ = 0.44) with 89% for both sensitivity and specificity. For the test-set (*N* = 15), the model correctly classified 80% of patients, with a sensitivity and specificity of 87 and 83%, respectively. From the NCS-R*_*time–*1_* training scores (*N* = 28), 86% of the patients were correctly classified (Homer-Lemeshow *p* = 0.48; Cox & Snell R^2^ = 0.40) with a sensitivity of 100% and a specificity of 80%. In testing (*N* = 15), 60% of patients were correctly classified, with 67% sensitivity and 58% specificity (Table 5).

**TABLE 5:** Confusion matrix and classification’s results by logistic regression in training and validation test.

		Logistic regression Training test *N* = 28 (−6.403 + 1.447×NCS)		Logistic regression Validation test *N* = 15		Logistic regression Training test *N* = 28 (−5.569 + 1.747×NCS-R)		Logistic regression Validation test *N* = 15	
		NCS		NCS-R		NCS		NCS-R	
		VS/UWS → MCS	VS/UWS → VS/UWS°	VS/UWS → MCS	VS/UWS → VS/UWS°	VS/UWS → MCS	VS/UWS → VS/UWS°	VS/UWS → MCS	VS/UWS → VS/UWS°
**Metrics**	**VS/UWS → MCS**	**17**	**1**	**7**	**1**	**18**	**0**	**7**	**1**
	**VS/UWS → VS/UWS°**	**2**	**8**	**2**	**5**	**4**	**6**	**5**	**2**

Bad prediction (%)		11		20		14		40	
Correct prediction (%)		89		80		86		60	
Precision (%)		94		87		100		87	
Negative predictive values (%)		80		71		60		29	
Sensitivity (%)		89		78		82		58	
Specificity (%)		89		83		100		67	
Accuracy (%)		89		80		86		60	
Balanced accuracy (%)		87		79		80		58	
positive Likelihood Ratio		8.1		4.7		-		1.8	
negative likelihood Ratio		0.1		0.3		0.2		0.6	

## Discussion

Despite the many tools available to evaluate conscious characteristics in DOC patients (Starmark et al., 1988; Shiel et al., 2000; Wijdicks et al., 2005), subtle differences between different DOC severities, particularly the VS/UWS and MCS conditions, can only be detected with more sensitive evaluations (Majerus et al., 2005). Moreover, variations in the environment, the examiner, and the state of a patient can lead to diagnostic error. Given the absence of communication abilities in DOC groups, evaluations based on overt behavior have been vital in establishing robust diagnostic guidelines (Monti et al., 2010).

Accordingly, the NCS(-R) was developed to clinically assess pain perception in UWS/VS and MCS patients. Apart from evaluating conscious characteristics, its administration was recommended because patients may already experience pain as a result of conditions related to their circumstances (e.g., polytrauma injuries, decubitus ulcers, bedsores, and spasticity) (Chatelle and Thibaut, 2014; Chatelle, 2016). Evaluating pain perception is also a very important factor to clinical decisions regarding treatment, such as the administration of drugs to relieve pain (analgesic treatment) (Chatelle and Thibaut, 2014). In addition to their potential usefulness, both the NCS and its revised version have been confirmed to be valid clinical tools to assess nociception (Vink et al., 2017).

Previously, a strong correlation was found between patients’ CRS-R scores and their responsiveness to noxious stimulation as measured by the NCS-R. More specifically, an NCS-R score of 2 or more was found to be the best threshold to indicate nociception and differentiate MCS from VS/UWS (Chatelle et al., 2018). However, in a previous attempt to link these measures with the recovery of VS/UWS patients, no correlation was found between the initial NCS-R assessment and whether a patient’s condition improved 6 months afterward (Bagnato et al., 2018).

To address this important prognostic problem, our study aimed to verify if changes to the scores of these nociceptive assessments can predict an improvement from VS/UWS to MCS. In our sample of DOC patients, we observed that 13 of the 17 VS/UWS individuals (76%) who were eventually diagnosed as MCS with the CRS-R showed significant behavioral changes with the NCS/NCS-R prior to reaching this stage. We also found threshold scores to describe this transition, which were equal to or higher than 3 for the NCS-R and 5 for the NCS (Table 3). Overall, the NCS and NCS-R did not show significant differences in performance (Table 3), confirming the results of Vink et al. (2017) on the validity of both scales.

With the NCS, the two different statistical approaches (logistic regression and predictive accuracy with the ROC curve) did not show significant differences in determining the probability of emerging out of VS/UWS. With the NCS-R, however, logistic regression produced a more accurate classification for patients who did not change in their CRS-R diagnosis, while the ROC curve’s threshold value was more accurate in classifying the patients who transitioned to MCS. Furthermore, only the NCS maintained high predictive accuracy, specificity, and sensitivity when logistic regression was applied in the training and validation procedure.

Overall, the relationship between CRS-R and NCS(-R) scores we report is in line with previous works assessing nociception in DOC populations. A study by Chatelle et al. (2018) reported that higher CRS-R total scores are associated with more appropriate behavioral responses in the NCS-R assessment. They also showed a strong correlation between the motor and oromotor sub-scores of CRS-R and the NCS-R total score.

In general, nociception relies on a wide brain network that is linked to conscious processing (Schnakers et al., 2010; Riganello et al., 2014; Chatelle and Laureys, 2015), and is therefore a very important factor to consider for DOC patients. Other works confirmed a correlation between NCS(-R) total scores and cortical metabolic measurements of brain regions related to nociception (Chatelle et al., 2014; Chatelle, 2016), underscoring the validity of these scales. Furthermore, a number of score cut-offs for these assessments were proposed to indicate values that may correspond to substantial pain perception, which is associated with the MCS condition (Chatelle et al., 2012, 2018; Vink et al., 2017).

Although the thresholds we found showed high predictive accuracies, the absence of visual scoring in the NCS-R seemed to increase the predictive error for patients who did not undergo this transition. The visual subscale was excluded from the NCS-R because no significant difference was found for it in noxious and non-noxious conditions (Chatelle and Thibaut, 2014). However, oriented movements toward visual stimuli tend to be one of the first signs of overt consciousness for patients emerging out of UWS/VS. Accordingly, 5 of the 17 (29%) subjects diagnosed as MCS with the CRS-R were better classified with the NCS than the NCS-R in the week prior, which appears to be the result of the visual subscale’s contribution.

Other studies on nociception in DOC populations took a different approach to investigating their capabilities. For example, Cortese et al. (2020) reported that patients who were diagnosed as VS/UWS and demonstrated no oriented or reflexive behavioral responses to noxious stimulation still showed a trace conditioning of the nociceptive stimulus. This was seen with a conditioning protocol where the conditioned (noxious) stimulus was presented, terminated, and followed by an unconditioned stimulus, such as music. The patients who demonstrated nociceptive trace conditioning evolved to MCS within 4 weeks. This finding underscored the difficulty of correctly diagnosing DOC conditions through behavior, which can be made even more difficult by external factors, such as drug therapy (Riganello et al., 2021).

Furthermore, given the subjective aspect of pain and the inability of VS/UWS patients to describe it, behavioral responses do not necessarily indicate the extent to which conscious processing is involved (Loeser and Treede, 2008). Even so, assessments based on pain remain essential for these patients, and it hence may be necessary to be more thorough with the administration of noxious stimulation (Chatelle and Thibaut, 2014; Naro et al., 2015; Schnakers and Zasler, 2015; Garcia-Larrea and Bastuji, 2018). For example, Formisano et al. (2020) proposed employing NCS(-R) with a range of personalized stimuli (e.g., hand opening, upper limb abduction, and head mobilization), which may present different responses compared to the simpler procedure of applying pressure to the fingernail bed.

Generally, higher scores in NCS(-R) scales are indicative of a more complex response to noxious stimuli and the level of conscious modulation that may be involved (Vink et al., 2017). The concept of a “Pain Matrix” is often used to explain the presence of a conscious experience related to a painful stimulus. Functional imaging studies found that in response to an experience that may induce nociception, global brain metabolism in VS/UWS patients differed from healthy subjects in two areas: the left insula and the Anterior Cingulate Cortex (ACC) (Bonin et al., 2019). DOC patients demonstrating residual metabolism in these areas in response to pain could have the potential for behavioral responsiveness, which is also supported by a correlation between higher ACC activation and increased pain perception in conscious, healthy volunteers (Bonin et al., 2019).

Considering these previous findings, our results could be supported by the idea that an increased NCS(-R) total score is correlated to higher cortical activity in DOC patients, despite a lack of oriented behaviors that the CRS-R evaluates.

In conclusion, our data support the general hypothesis that assessments based on noxious stimulation could help predict some level of recovery from severe disorders of consciousness. Our methodology showed how the NCS(-R) can complement the CRS-R in prognostic considerations. More specifically, our findings show that VS/UWS patients who reach total scores ≥ 5 for the NCS or ≥3 for the NCS-R are at a higher likelihood of regaining some level of consciousness and attaining an MCS diagnosis with the CRS-R. We note that while the CRS-R can effectively differentiate MCS and VS/UWS conditions, the NCS(-R) cannot meet this purpose. However, this work highlights that it is still useful in identifying nociceptive processing, which could provide valuable prognostic information about VS/UWS patients. In terms of behavioral responses to noxious stimuli, the NCS(-R) provides even more information on nociception than the CRS-R, which underscores its value in making clinical decisions.

Although these results are very promising, the limited patients’ sample, different aetiologies, and wide age range of our population sample could present limitations to this work. Further investigations with our data and other patient samples may be needed to confirm our findings.

## Data Availability Statement

The original contributions presented in the study are included in the article/supplementary material, further inquiries can be directed to the corresponding author/s.

## Ethics Statement

The studies involving human participants were reviewed and approved by Ethic Committee of “Regione Calabria Comitato Etico Sezione Area Centro” of Catanzaro. The patients/participants provided their written informed consent to participate in this study.

## Author Contributions

MC: concept and design. MC and FA: acquisition and assessment. FR: statistical analysis. FR, MC, and FA: interpretation of data. FR, MC, FA, and IN: drafting of the manuscript. AS: critical revision of the manuscript. All authors: final revision of the manuscript.

## Conflict of Interest

The authors declare that the research was conducted in the absence of any commercial or financial relationships that could be construed as a potential conflict of interest.

## Publisher’s Note

All claims expressed in this article are solely those of the authors and do not necessarily represent those of their affiliated organizations, or those of the publisher, the editors and the reviewers. Any product that may be evaluated in this article, or claim that may be made by its manufacturer, is not guaranteed or endorsed by the publisher.

## Footnotes

1
https://www.wma.net/what-we-do/medical-ethics/declaration-of-helsinki/

2
www.wagnerinstruments.com
